# Gene Expression Profiles of Colonic Mucosa in Healthy Young Adult and Senior Dogs

**DOI:** 10.1371/journal.pone.0012882

**Published:** 2010-09-22

**Authors:** Dong Yong Kil, Brittany M. Vester Boler, Carolyn J. Apanavicius, Lawrence B. Schook, Kelly S. Swanson

**Affiliations:** 1 Department of Animal Sciences, University of Illinois, Urbana, Illinois, United States of America; 2 Division of Nutritional Sciences, University of Illinois, Urbana, Illinois, United States of America; 3 Department of Veterinary Clinical Medicine, University of Illinois, Urbana, Illinois, United States of America; Duke University, United States of America

## Abstract

**Background:**

We have previously reported the effects of age and diet on nutrient digestibility, intestinal morphology, and large intestinal fermentation patterns in healthy young adult and senior dogs. However, a genome-wide molecular analysis of colonic mucosa as a function of age and diet has not yet been performed in dogs.

**Methodology/Principal Findings:**

Colonic mucosa samples were collected from six senior (12-year old) and six young adult (1-year old) female beagles fed one of two diets (animal protein-based vs. plant protein-based) for 12 months. Total RNA in colonic mucosa was extracted and hybridized to Affymetrix GeneChip® Canine Genome Arrays. Results indicated that the majority of gene expression changes were due to age (212 genes) rather than diet (66 genes). In particular, the colonic mucosa of senior dogs had increased expression of genes associated with cell proliferation, inflammation, stress response, and cellular metabolism, whereas the expression of genes associated with apoptosis and defensive mechanisms were decreased in senior vs. young adult dogs. No consistent diet-induced alterations in gene expression existed in both age groups, with the effects of diet being more pronounced in senior dogs than in young adult dogs.

**Conclusion:**

Our results provide molecular insight pertaining to the aged canine colon and its predisposition to dysfunction and disease. Therefore, our data may aid in future research pertaining to age-associated gastrointestinal physiological changes and highlight potential targets for dietary intervention to limit their progression.

## Introduction

The primary role of the colon has been known for years to maintain water and electrolyte balance and to excrete undigested food materials. Currently, the colon is appreciated as a metabolically active organ and, therefore, colonic health is closely linked with overall health of humans and animals [1]. Until recently, however, the physiology of the colon has received little attention in biological studies as compared to other body organs.

Dietary composition may be the most important factor affecting colonic health because of its direct effects on microbial fermentation, morphology, and metabolism. Aging also plays a significant role in colon health. It is well known that age is highly associated with an increased risk of colonic diseases in humans [2], [3]. Similarly, dogs become more susceptible to gastrointestinal disorders with age [4]. Our previous experiment reported significant differences in colonic butyrate concentrations and morphology (e.g., crypt depth) between senior and young adult dogs [5]. However, the molecular mechanisms underlying the effects of age and diet on colonic physiology remain unstudied.

Gene expression profiling may improve our understanding of colonic physiology and metabolic alterations as a function of age and diet. The PCR and Northern-blotting assays have been widely used for measuring gene expression changes in humans and animals for years; however, they are only capable of monitoring a limited number of genes at a time. As a powerful alternative to those classical methods, DNA microarrays can analyze thousands of genes simultaneously, providing a global view of gene expression [6]. In recent years, microarrays have been adopted to investigate how genes are differentially expressed in diseased individuals [7], in response to dietary treatments [8], and according to physiological stage [9], [10]. Therefore, microarrays may be used to link molecular events with physiological response and identify critical genes and biological pathways.

Previously, we reported the effects of diet (APB; animal protein-based vs. PPB; plant protein-based) and age (young adult vs. senior dogs) on gene expression profiles of cerebral cortex [11], skeletal muscle [12], and abdominal adipose tissues [13]. To our knowledge, no large-scale molecular analysis of colonic mucosa in young adult vs. senior dogs is available. Therefore, we isolated RNA from colonic mucosa that was collected from the experiment of Kuzmuk et al. [5] and measured gene expression profiles using commercial microarrays. The objective of this experiment, therefore, was to compare colonic mucosal gene expression in healthy young adult vs. senior dogs fed two distinct diets.

## Results and Discussion

The characteristics of undigested food residue, microbial populations, and their fermentation play a key role in colonic health and physiology [14]. In our previous experiments [5], [15], it was demonstrated that age and diet influenced nutrient digestibility, intestinal morphology, and colonic fermentation patterns in dogs. In short, senior dogs had greater (P<0.05) apparent total tract digestibility of organic matter and fat as compared to young growing dogs, while these differences were undetectable as young dogs became mature (∼12 month old). Dogs consuming PPB had a lower (P<0.01) fat digestibility, tended to have decreased (P<0.10) organic matter (OM) digestibility, but had increased (P<0.01) crude protein (CP) digestibility than dogs consuming APB. Senior dogs had deeper (P<0.01) colonic crypt depth and greater (P<0.05) colonic concentrations of butyrate compared with young adult dogs regardless of diets. Dogs consuming APB had greater (P<0.05) colonic concentrations of ammonia and butyrate than dogs consuming PPB. These observations bring to question the relationship between physiological response and colonic transcriptional activity as a function of age and diet. To address this question, we used DNA microarray technology to provide a global view of gene expression and advance our understanding of the molecular events occurring in colonic mucosa.

### Global alterations in gene expression due to age and diet

**Figure 1:**
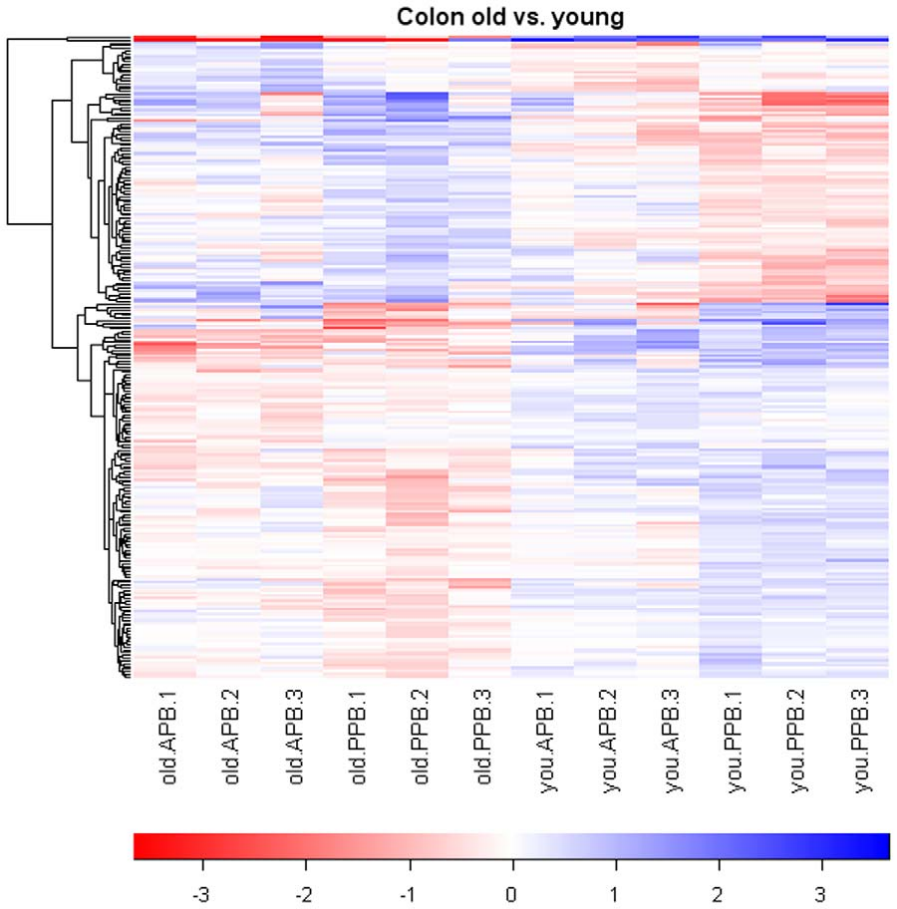
Heatmap of senior vs. young adult dog pairwise comparisons. Values are the GCRMA-processed probe set value (Log_2_ scale) minus the mean value for that probe set across all arrays. The dendrogram was created by hierarchical cluster analysis.

**Figure 2:**
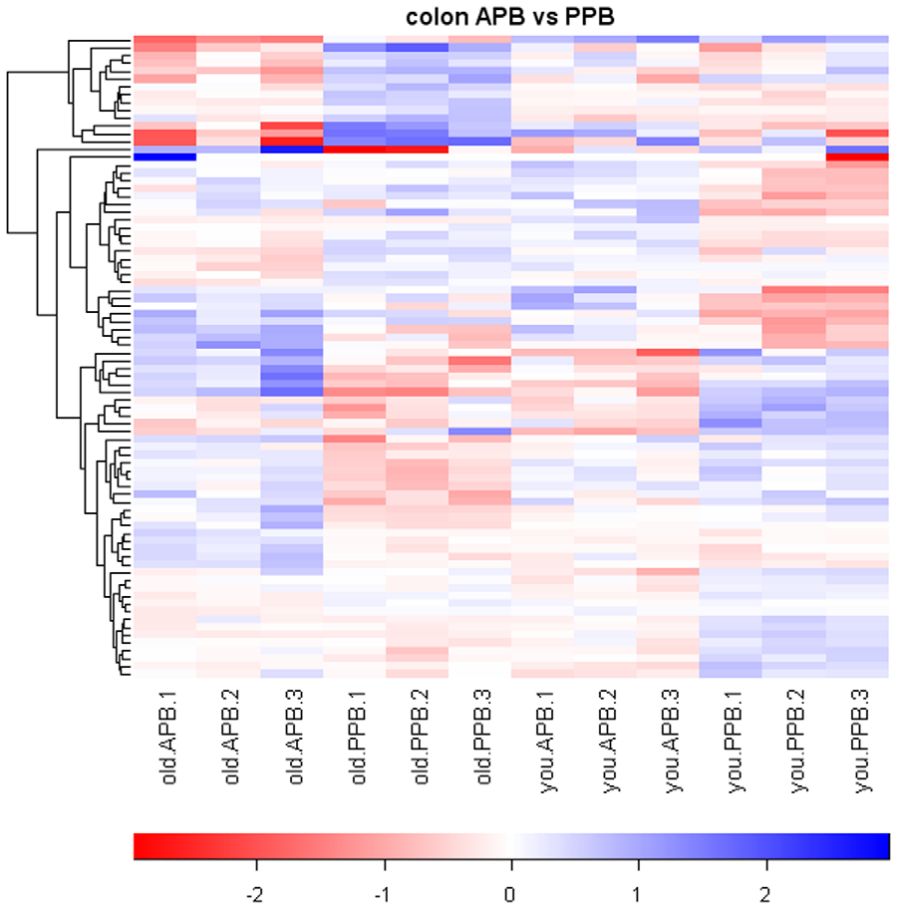
Heatmap of animal-protein based diet (APB) vs. plant-protein based diet (PPB) pairwise comparisons. Values are the GCRMA-processed probe set value (Log_2_ scale) minus the mean value for that probe set across all arrays. The dendrogram was created by hierarchical cluster analysis.

A total of 278 gene transcripts were identified as being differentially expressed by age (212 genes) and/or diet (66 genes), according to the pre-planned statistical screening methods (Table 1). The magnitude and patterns of gene expression due to age and diet are presented in Fig. 1 and Fig. 2, respectively. The heat map comparing young adult and senior dogs displayed relatively consistent alterations in gene expression due to age regardless of diet, while a lack of consistency is demonstrated by the heat map comparing dietary treatments.

**Table 1:** Global view of colonic mucosal gene expression alterations in senior vs. young adult dogs fed an animal protein-based diet (APB) or plant protein-based diet (PPB).

	Number of gene transcripts changed^1^	Number of annotated genes changed^2^
Total genes differentially expressed	278 (1.96%)	138
Age-associated alterations	212 (1.49%)	106
Up-regulated	84	45
Down-regulated	128	61
Diet-associated alterations	66 (0.46%)	32
Up-regulated	31	19
Down-regulated	35	13

1 Values in the parenthesis represent the percentage of gene transcripts differentially expressed in relation to the total number of genes expressed in colon tissue (14,217 genes).

2 Number of annotated and non-redundant genes that had >1.5 fold-change in gene expression.

The fact that most gene expression differences were attributed to age in this experiment agrees with our previous experiments focused on gene expression in cerebral cortex [11], skeletal muscle [12], and abdominal adipose [13] tissues from these same dogs. Of the 14,217 genes expressed by colon mucosa, 1.49% (212/14,217) were significantly altered due to age. Our previous experiments also reported age as the primary factor affecting gene expression changes, with a small percentage of genes being altered. In short, only 0.25% of genes in adipose tissue [13], 2.91% of genes in skeletal muscle [12], and 6.48% of genes in cerebral cortex [11] were differentially expressed due to age. The small number of genes altered by age in this experiment is in agreement with previous microarray data from muscle [9], duodenum, and colon [16] tissues of aged vs. young mice. Taken together, these experiments suggest that age-associated physiological changes in body tissues are mediated by the transcriptional alteration of a small number of genes and these changes are tissue-specific [9].

One unique quality of colonic tissue vs. those listed above is that it may be greatly impacted by intestinal microbiota. Microbes may directly affect colonic epithelia via contact or production of numerous secretions (e.g., bacteriocins), but may also have an indirect impact through the fermentation of carbohydrate- and protein-containing substrates in the colon, both of which may lead to gene expression responses. Although dogs do not rely on microbial fermentation as a significant source of energy as in ruminant or large herbivorous species, a stable population of intestinal microbes is crucial to host health. Because intestinal microbial populations change with increased age in dogs, even in those that are healthy, their impact on the gene expression profiles measured in this study cannot be discounted. Microbial populations were not analyzed in this experiment, but would be worth measuring in future studies of this kind.

Diet affected the expression of a very small percentage (0.46%) of genes (66/14,217) in the current experiment. This finding also agrees with our previous observations [11], [12], [13]. However, age-associated gene expression alterations were somewhat dependent on diet, as noted in Tables 2–6 and Figure 2. For instance, the number of gene expression changes due to age was greater for dogs consuming PPB (87 genes) compared with dogs consuming APB (24 genes) in this experiment. This observation may be a consequence of greater dietary fiber and fermentative activity in dogs fed PPB, as they have been known to influence colon physiology, including colonic microbiota, fermentative by-products, and morphology. While the concentrations of colonic total short-chain fatty acids (µmol/g dry matter) were not different in dogs fed the APB and PPB diets [5], total dry fecal output of dogs fed PPB was approximately 1.7-fold higher than in dogs fed APB [15]. Therefore, the total short-chain fatty acids measured in colonic contents of dogs fed PPB was nearly twice that of dogs fed APB. This difference in organic acid production, or potential differences in intestinal microbiota due to diet may have influenced our results.

**Table 2:** Up-regulated cell growth and development- and cellular metabolism and protein processing-associated genes in colonic mucosa of senior vs. young adult dogs.

			Fold change	
Functional classification	Gene name	Gene symbol	APB	PPB
**Cell growth and development**				
Wound healing	Connective tissue growth factor	CTGF		3.49
Proliferation	RAB GTPase activating protein 1-like	RABGAP1L	2.37	
Proliferation	Retinoic acid receptor responder 1	RARRES1		3.49
Proliferation	Bone morphogenetic protein 6	BMP6		2.23
Proliferation	MAP/microtubule affinity-regulating kinase 1	MARK1		2.21
Proliferation	SMAD family member 5	SMAD5	2.08	3.46
Proliferation	Tribbles homolog 1	TRIB1		2.07
Proliferation	Potassium channel tetramerisation domain containing 10	KCTD10		2.01
Proliferation	FBJ murine osteosarcoma viral oncogene homolog	FOS		1.92
Structure	Fig4 homolog	FIG4	1.71	
Structure	Filamin B, beta	FLNB		7.63
Apoptosis	Reticulon 3	RTN3		1.60
**Cellular metabolism and protein processing**				
ATP synthesis	ATPase, Ca transporting, plasma membrane 1	ATP2B1	1.56	
Electron transport	Cytochrome c oxidase polypeptide VIa-heart, mitochondrial precursor	COXVIAH		6.21
TCA cycle	Dihydrolipoamide S-succinyltransferase	DLST		6.17
Cu metabolism	Ceruloplasmin	CP		3.77
One-carbon metabolism	Methionine adenosyltransferase II, alpha	MAT2A		2.41
Lipid metabolism	Carnitine palmitoyltransferase II	CPT2		1.56
Folate synthesis	Methenyltetrahydrofolate synthetase domain containing	MTHFSD		1.83
Protein binding	Ring finger protein 103	RNF103	1.92	
Protein binding	Armadillo repeat containing, X-linked 3	ARMCX3		2.46
Protein transport	Eukaryotic translation initiation factor 4E nuclear import factor 1	EIF4ENIF1		2.28
Protein transport	Solute carrier family 1 (glutamate/neutral amino acid transporter), member 4	SLC1A4		1.61
Protein localization	Vacuolar protein sorting 13D isoform 1	VPS13D		5.71
Protein phosphorylation	PCTAIRE protein kinase 1	PCTK1		2.04
Protein acylation	Glutamyl-prolyl-tRNA synthetase	EPRS		1.93
Proteolysis	Potassium channel modulatory factor 1	KCMF1		2.09
Protein depalmitoylation	Palmitoyl-protein thioesterase 1	PPT1		5.35
Endosome transport	Early endosome antigen 1	EEA1	3.44	
Membrane traffic	SEC16 homolog A	SEC16A		2.74

**Table 3 pone-0012882-t003:** Up-regulated cell signaling and signal transduction-, immune and stress response-, and transcription/translation-associated genes in colonic mucosa of senior vs. young adult dogs.

			Fold change	
Functional classification	Gene name	Gene symbol	APB	PPB
**Cell signaling and signal transduction**				
Signal transduction	WD repeat domain, phosphoinositide interacting 1	WIPI1		1.78
Signal transduction	Sel-1 suppressor of lin-12-like	SEL1L		1.71
Signal transduction	Guanine nucleotide binding protein (G protein), beta polypeptide 1	GNB1		1.77
**Immune and stress response**				
Nitric oxide synthesis	Heat shock protein HSP 90-alpha (HSP 86)	HSP90AA1		3.82
Immune response	Immunoglobulin lambda-like polypeptide 1	CD179b		1.85
**Transcription-Translation**				
Transcription	Myosin 1C	MYO1C		1.90
Translation	Eukaryotic translation initiation factor 5B	EIF5B	2.50	
Translation	Mitochondrial methionyl-tRNA formyltransferase	MTFMT	2.40	
RNA processing	Splicing factor, arginine/serine-rich 5	SFRS5		3.56
RNA processing	Helicase with zinc finger	HELZ		2.47
RNA processing	Fusion (involved in t(12;16) in malignant liposarcoma)	FUS		1.88
**Miscellaneous and unknown**				
Muscle development	Myosin, light chain 6, alkali, smooth muscle and non-muscle	MYL6		3.06
Unknown	Golgi membrane protein 1	GOLM1		3.55
Unknown	Fibronectin type III domain containing 3B	FNDC3B		2.50
Unknown	Transmembrane protein 205	TMEM205		1.98

**Table 4 pone-0012882-t004:** Down-regulated cell growth and development- and cell signaling and signal transduction-associated genes in colonic mucosa of senior vs. young adult dogs.

			Fold change	
Functional classification	Gene name	Gene symbol	APB	PPB
**Cell growth and development**				
Apoptosis	Somatostatin	SST	−20.08	−7.43
Apoptosis	Programmed cell death 2	PDCD2		−2.03
Differentiation	Deleted in malignant brain tumors 1 isoform c	DMBT1	−83.88	
Differentiation	Cysteine and glycine-rich protein 2	CSRP2		−1.62
Proliferation	Preproglucagon	GCG	−3.20	−3.22
Proliferation	Polyamine-modulated factor 1	PMF1	−1.65	
Cell cycle	Protein phosphatase 2, regulatory subunit B′, gamma isoform	PPP2R5C		−2.34
Cell cycle	Septin 4 isoform 2	SEPT4		−1.92
Structure	Rho GDP dissociation inhibitor (GDI) beta	ARHGDIB	−2.99	
**Cell signaling and signal transduction**				
Neuroendocrine signaling	Secretogranin 1/chromogranin B	CHGB	−6.07	−2.20
Neuroendocrine signaling	Secretogranin V	SCG5	−1.83	
Neuroendocrine signaling	Secretogranin III	SCG3		−2.97
Signal transduction	Diacylglycerol kinase zeta	DGKZ	−2.25	
Signal transduction	ADP-ribosylation factor-like 6	ARL6		−2.34
Signal transduction	Regulator of G-protein signaling 10	RGS10		−1.98
Signal transduction	Cornichon homolog 4	CNIH4		−1.89
Signal transduction	Rho GTPase activating protein 15	ARHGAP15		−1.85
Signal transduction	TYRO protein tyrosine kinase binding protein	TYROBP		−1.77
Neurotransmission	Synaptosomal-associated protein, 25kDa	SNAP25		−2.07

**Table 5 pone-0012882-t005:** Down-regulated cell metabolism and protein processing-associated genes in colonic mucosa of senior vs. young adult dogs.

			Fold change	
Functional classification	Gene name	Gene symbol	APB	PPB
**Cellular metabolism and protein processing**				
Glucose metabolism	Glucokinase (hexokinase 4) regulator	GCKR		−8.32
Folate metabolism	Folate hydrolase 1	FOLH1		−3.16
CO_2_ metabolism	Carbonic anhydrase II	CA2	−2.96	
Cellular respiration	CDGSH iron sulfur domain 1	CISD1		−2.11
Arginine metabolism	Argininosuccinate lyase	ASL	−1.83	
Nucleic acid metabolism	Nucleoside diphosphate kinase 4	NEM4	−1.58	
Nucleic acid metabolism	Deoxyguanosine kinase	DGUOK		−1.53
Lipid metabolism	Peroxisomal trans-2-enoyl-CoA reductase	PECR		−1.99
Sulfur metabolism	Molybdenum cofactor synthesis 2	MOCS2		−1.87
Hydrolysis	N-terminal asparagine amidase	NTAN1		−1.73
Electron transport chain	NADH dehydrogenase (ubiquinone) 1 beta subcomplex, 9, 22kDa	NDUFB9		−1.53
Protein transport	RAB9A, member RAS oncogene family	RAB9A		−1.69
Protein binding	Receptor-interacting factor 1 isoform 1	C1orfl03		−2.93
Protein binding	Bridging integrator 2	BIN2		−2.16
Protein binding	Makorin ring finger protein 1	MKRN1		−1.66
Protein folding	Serologically defined colon cancer antigen 10	SDCCAG10		−1.52
Protein modification	Phosphatidylinositol glycan anchor biosynthesis, class H	PIGH	−1.61	
Protein Ubiquitination	WD repeat, sterile alpha motif and U-box domain containing 1	WDSUB1		−1.89
Methylation	Methyltransferase like 5	METTL5		−2.08
Protein palmitoylation	Zinc finger, DHHC-type containing 2	ZDHHC2		−1.62

**Table 6:** Down-regulated immune and stress response- and transcription/translation-associated genes in colonic mucosa of senior vs. young adult dogs.

			Fold change	
Functional classification	Gene name	Gene symbol	APB	PPB
**Immune and stress response**				
Immune response	Lectin, galactoside-binding, soluble, 3	LGALS3	−3.02	
Immune response	Serum amyloid A1	SAA1		−1.91
Immune response	CD48 molecule	CD48		−1.88
Oxidative stress response	Glutathione peroxidase 7	GPX7		−2.11
Detoxification	Glutathione S-transferase M3 (brain)	GSTM3	−4.04	−8.69
**Transcription-Translation**				
Translation	Cytokine induced protein 29 kDa	SARNP	−1.69	
Translation	Mitochondrial ribosomal protein S28	MRPS28		−1.98
Translation	Mitochondrial ribosomal protein L21	MRPL21		−1.96
Translation	Phosphoseryl-tRNA kinase	PSTK		−1.54
Transcription	Transcription factor-like 5 protein	TCFL5		−2.92
Transcription	CBF1 interacting corepressor	CIR1		−1.70
DNA repair	General transcription factor IIH, polypeptide 4, 52kDa	GTF2H4	−1.63	
RNA processing	U7 snRNA-associated Sm-like protein	LSM11		−1.71
RNA processing	Splicing factor 3B, 14 kDa subunit	SF3B14		−1.65
**Miscellaneous and unknown**				
Reproduction	UBX domain protein 8	UBXN8		−1.99
Insulin secretion	Family with sequence similarity 3, member B	FAM3B		−1.63
Unknown	CE5 protein	CE5		−2.71
Unknown	Leucine zipper transcription factor-like 1	LZTFL1		−2.19
Unknown	Glyoxalase domain containing 5	GLOD5		−2.08
Unknown	Ribonuclease H2, subunit B	RNASEH2B		−1.68
Unknown	Leydig cell tumor 10 kDa protein homolog	C19orf53		−1.66
Unknown	STARD3 N-terminal like	STARD3NL		−1.56

Following removal of unannotated genes, duplicate probe sets of the same gene, and genes that had <1.5 fold-change in expression, 138 genes were identified as being differentially expressed due to age (106 genes) and/or diet (32 genes). Quantitative real-time PCR (qRT-PCR) was subsequently used to further validate the responses of age and diet on the selective genes of interest using the method of Vester et al. [17]. A majority of the comparisons (8/11 genes) via qRT-PCR were in agreement with the microarray data (data not shown).

### Age affected genes associated with cell growth and development

Of the 106 genes differentially expressed due to age, 45 were up-regulated (Tables 2 and 3) and 61 were down-regulated (Tables 4–6). In particular, senior dogs had an up-regulation of tumorigenic genes and down-regulation of anti-tumorigenic genes in this experiment. This finding is in agreement with a previous experiment suggesting that aged mice are predisposed to colon cancer because of an up-regulation of cancer-associated genes [16]. Although dogs do not develop colon cancer at high rates [18], these alterations in gene expression may be indicative of the increased risk of other colonic diseases with age in dogs.

Connective tissue growth factor (CTGF), a gene associated with wound-healing [19], was up-regulated (3.49 fold) in senior dogs consuming PPB, which may indicate increased cellular damage or need for epithelial repairs in the aged colon. High CTGF expression was found in benign colorectal tumors, while metastatic colorectal tumors had a low CTGF expression [20]. Up-regulated CTGF expression has also been implicated in various human cancers such as those affecting the esophagus [21], prostate [22], and breast [23]. Up-regulated retinoid acid receptor responder 1 (RARRES1), as observed in senior dogs consuming PPB, has been suggested to be present during the early stage of adenoma and tumor progression [24]. The FLNB gene related to cellular communication between actin networks and cell membrane [25] was up-regulated (7.63 fold) in senior dogs consuming PPB. The mutation in FLNB has displayed defects in skeletal and vascular development [26], but little is known about its function in colonocytes. It is interesting to note that these gene expression changes only occurred in senior dogs consuming the PPB diet, suggesting an interaction effect. While it is only speculative, it is possible that the increased fermentative action by microbes and/or energy available from the resulting short-chain fatty acids, which is known to increase intestinal epithelial cell metabolism and proliferation, was necessary to highlight these age-related differences in gene expression.

Aged colon in this experiment appeared to have an altered activity of the Wnt signaling pathway, as many genes related to that pathway, including MARKL1 [27], BMP6 [28], SMAD5 [29], and RABGAP1L [30], were up-regulated in senior vs. young adult dogs. The Wnt signaling pathway, which consists of several genes affecting cellular proliferation, differentiation, and apoptosis, is thought to regulate growth and renewal of the colorectal epithelium [31]. The activity of β-catenin, which is an important transcriptional factor in Wnt signaling, is suggested to be increased during epithelial proliferation and differentiation in colonic crypts [31]. Increased expression of SMAD5, a transcriptional factor associated with the TGF-β-SMAD signaling pathway, was observed in senior dogs consuming PPB or APB. The TGF-β-SMAD signaling pathway seems to have a contrasting role in colonic epithelium as a tumor suppressor in normal cells and as a tumor enhancer in cancer cells [32]. The FOS gene, a mediator in TGF-β-SMAD signaling pathway [33], was also differentially expressed in the aged canine colon. The significance of altered TGF-β-SMAD signaling in the aged colon is unclear. Our data also suggest that the MAP kinase pathway, which is associated with cell differentiation, proliferation, and apoptosis [34], was up-regulated in aged dogs. The expression of TRIB1, a potential regulator of this pathway [35], was up-regulated (2.07 fold) in aged colonic mucosa. Collectively, an increased activity of genes associated with cell proliferation and growth was observed in aged colon tissue. These genes and pathways may explain, in part, our previous observation of greater colonic crypt depth in senior dogs [5]. However, because the Wnt, BMP, and TGF-β-SMAD pathways are highly complex and interact with one another, further experiments are required to determine the implications of these pathways as it pertains to intestinal health of senior dogs.

In line with greater crypt depth, several genes associated with induction of apoptosis and inhibition of tumor growth were down-regulated in the aged colon. Deleted in malignant brain tumor-1 (DMBT-1) was down-regulated by approximately 84-fold in senior dogs consuming APB. The DMBT1 gene, a mucin-like glycoprotein, is considered a potential tumor-suppressor gene and its down-regulation has been implicated in human esophageal, gastric, and colorectal cancer [36]. In addition, the down-regulation of DMBT1 has also been associated with decreased mucosal immune response and epithelial differentiation in the lung and intestine [37]. Somatostatin (SST), a neuropeptide acting on the central nervous system and gastrointestinal tract, was greatly down-regulated in senior dogs consuming APB (20.08 fold) or PPB (7.43 fold). The SST gene is thought to inhibit tumor growth through the suppression of growth-promoting factors, cell cycle arrest, and an induction of apoptosis [38]. In addition to its anti-tumor effects, SST plays a role in maintaining intestinal homeostasis by regulating the secretion of intestine-associated hormones (e.g., pancreatic polypeptide) and intestinal motility [39].

The RhoGDI2 (ARHGDIB) gene was down-regulated (2.99 fold) in senior dogs consuming APB. This gene inhibits the activation of Rho family protein regulating cellular structure, adhesion, and motility, and is thought to be a metastasis suppressor gene [40]. The decreased expression of PPP2R5C, a regulatory subunit of protein phosphatase 2A, in senior dogs consuming PPB also suggests a decreased ability to prevent tumor growth in the aged colon. Recent evidence has demonstrated that PPP2R5C-protein phosphatase 2A complex activates p53 and plays an additive role in the tumor suppressing effects of p53 in response to DNA damage [41]. Interestingly, expression of PMF1, a transcriptional factor to induce spermidine/spermine N1-acetlytransferase that is critical for polyamine catabolism [42], was down-regulated (1.65 fold) in the aged colon. Polyamine is essential for cell proliferation and repair [43]. It is possible that the decreased expression of PMF1 may help to maintain or increase polyamine concentrations in the aged colon by retarding the polyamine catabolic rate. Therefore, down-regulation of PMF1 may be another mechanism by which greater crypt depth was observed in senior vs. young adult dogs.

The down-regulation of pre-proglucagon (GCG) in the aged colon was unexpected. The pre-proglucagon is a zymogen that is eventually processed by post-translational modification into glucagon, glucagon-like peptide (GLP)-1, GLP-2, or oxymodulin [44]. It is known that GLP-1 inhibits gastric acid secretion [45] and intestinal motility [46], while GLP-2 stimulates intestinal cell proliferation and differentiation with a concomitant reduction in apoptosis [47], [48]. Therefore, the down-regulation of GCG, as observed in senior dogs, may imply impaired intestinal function and decreased proliferative activity of epithelial cells in the aged colon. Likewise, we observed the down-regulation of other genes associated with cell proliferation and differentiation (CSRP2) and cell cycle and cytokinesis (SEPT4). The reason for varied expression of genes associated with cell proliferation, differentiation, and apoptosis is unclear. Taken together, we speculate that the altered expression of genes involved in cellular apoptosis, differentiation, and proliferation, concurrent with the decreased expression of genes involved in intestinal hormone secretion and motility (SST and GCG), may contribute to the predisposition of intestinal dysfunction and disease in the aged colon.

### Age affected genes associated with immunity and stress response

The colon is arguably the most immunologically active and stressed organ because of its continuous challenge from an environment rich in microbiota and its metabolites [49]. It is well known that a chronic inflammatory state predisposes the host to various colonic diseases [50]. The expression of several genes related to stress and inflammatory response were altered in senior vs. young adult dogs in this experiment. Heat shock protein (HSP90AA1), which is a molecular chaperone and works with nitric oxide synthase (NOS) to produce nitric oxide (NO) [51], was up-regulated (3.82 fold) in the aged colon. Nitric oxide is an important signaling molecule associated with normal body homeostatic functions; however, a surplus of NO is closely related to various pathological processes including carcinogenesis and inflammation [52]. It has been reported that increased NO production because of increased NOS activity occurs during chronic inflammation in the intestine [53], [54]. The up-regulation of HSP90AA1 has also been associated with the progression of gastric cancer [55]. Although colonic NOS activity was not measured in this experiment, it may be a useful measure in future aging studies.

Expression of CD179b, which is a premature antigen receptor in pre-B cells but is absent in mature B cells [56], was up-regulated in the aged colon. Interestingly, a down-regulation (3.02 fold) of galectin 3 (LGALS3) was observed in senior dogs consuming APB. It is noted that LGALS3 improves innate immune response during acute inflammation and plays a role in wound-healing during chronic inflammation [57]. The expression of SAA1, an acute phase protein and potent chemoattractant for monocytes and neutrophils [58], was also down-regulated in the aged colon. In colonocytes, SAA1 has a protective role in pathogenic invasion and cellular oxidative damage [59]. Moreover, we observed a down-regulation of GPX7 that encodes glutathione peroxidase (GTP) and GSTM3 that encodes glutathione-S-transferase (GST) in aged colonic mucosa. Glutathione peroxidase is known to neutralize lipid hydroperoxides and free hydrogen peroxides [60]. Glutathione-S-transferase is thought to eliminate cellular toxins such as xenobiotics, carcinogens, and lipid peroxides and to play a role in the prevention of colorectal cancer [61].

Although our old dogs were clinically healthy, our observations indicate that the aged colon appears to have an increased risk of inflammation and a decreased functionality of cellular defensive mechanisms. Our gene expression data are not surprising, as these responses may be expected in aged animals. However, they appear to contradict the increased luminal butyrate concentrations observed in senior vs. young adult dogs [5], as butyrate has been suggested to have therapeutic effects on colonic inflammation and cellular damage [62]. Butyrate is critical to the health of the colon, as it is the preferred energy substrate by colonocytes. Differences in butyrate utilization by colonocytes as a function of age may explain this equivocal finding. It has been reported that butyrate uptake in the colonic epithelium was reduced with age in rats [63]. We observed a down-regulation of genes associated with butyrate uptake, including carbonic anhydrase (CA2) that is required for the supply of hydrogen and bicarbonate [62] and somatostatin (SST) that may increase butyrate uptake by up-regulating monocarboxylate transporter 1 (MCT1) expression [64]. These observations support the notion that aged colon tissue may have a decreased ability to transport butyrate. Butyrate has been shown to enhance expression of GST, which suggests that some of the beneficial effects of butyrate on colonic health may be mediated by GST-related functions [65], [66]. Decreased GST expression in senior dogs further supports this hypothesis. Therefore, we speculate that our previous observation for increased luminal butyrate concentrations in aged dogs [5] may have been a consequence of impaired uptake and/or utilization of butyrate.

### Age affected genes associated with cellular metabolism

In aged colonic mucosa, there was increased expression of genes associated with ATP synthesis (ATP2B1), electron transport chain (COXVIAH), TCA cycle (DLST), and lipid oxidation (CPT2). These results are in agreement with previous reports of increased metabolic activity in the aged colon of rats fed ad libitum [16]. However, it should be noted that the number of genes involved and magnitude of gene expression associated with cellular metabolism was much larger in the previous experiment [16]. A possible reason for this discrepancy may be related to feeding methodology. A restricted feeding method, to maintain body weight, was used for senior dogs in this experiment, which may have attenuated some of the effects of age on gene expression associated with colonic metabolism, as caloric restriction generally decreases overall metabolic rate [9], [67]. Increased metabolic activity in the aged canine colon may have been a result of increased stress response and cell proliferation, as observed in this experiment, both of which require large amounts of energy. Increased crypt depth in senior dogs, as observed in our previous experiment, may be partly explained by an increased metabolic rate in the aged colon. The reason for the considerable down-regulation (8.32 fold) in glucokinase regulator (GCKR) in senior dogs consuming PPB is unknown, as this gene product regulates the activity of glucokinase that is primarily found in liver and pancreatic β-cells [68]. In this experiment, it is particularly interesting that senior dogs consuming PPB had a decreased expression (3.16 fold) of folate hydrolase (FOLH1). Folate hydrolase is required for intestinal folate uptake and its polymorphism has been linked to the development of colorectal cancer [69]. A previous experiment demonstrated that colonic folate absorption decreased with age and folate deficiency has been considered a risk factor for colon carcinogenesis in geriatrics [70]. Therefore, we speculate that the decreased folate absorption with increasing age is, in part, due to a reduction of folate hydrolase expression, as observed in this experiment. Decreased folate absorption may also be associated with increased expression (2.40 fold in dogs fed PPB) of methenyltetrahydrofolate synthetase (MTHFSD), which is involved in the folate biosynthetic pathway.

Although there was no consistent age-associated trend in expression of genes involved in protein processing, cellular signaling, or transcription-translation in this experiment, some genes of interest were differentially expressed. The VPS13D gene that was up-regulated (5.71 fold) in the aged colon has been associated with cellular vesicle-mediated sorting and protein transport [71]. Senior dogs had increased expression of PPT1 (palmitoyl protein thioesterase 1), a lysosomal enzyme that depalmitoylates proteins, and is reported to decrease TNF-induced apoptosis [72]. The expression of genes related to secretogranins (CHGB, SCG3, and SCG5) was decreased in senior dogs. Secretogranins/chromogranins are associated with the sorting and aggregation of secretory granules and are highly expressed in endocrine and neuronal cells [73]. The significance of age-related alterations in those genes is unclear at this time, but deserves attention in future experiments.

### Diet-associated alterations in gene expression

There was a small number of genes (32 genes) differentially expressed between dogs consuming APB and PPB (Tables 7 and 8). No consistent patterns of gene expression as a function of diet were also observed. The APB diet contained low dietary fiber and high animal protein concentrations, both of which have been implicated to have a negative impact on colonic health in humans [74], and were expected to result in greater differences. While colon cancer is not common in dogs as it is in humans, dogs often have diarrhea and/or increased production of putrefactive compounds (e.g., phenols, indoles, biogenic amines) and negative changes in intestinal microbiota populations in response to the consumption of high-protein diets or diets having low protein digestibility. The small sample size (n = 12), differences in daily caloric intake among dogs, and low to moderate fermentability of the dietary fiber in PPB, much of which was derived from corn and wheat middlings, may also have contributed to the lack of response in colon tissue. Finally, the short, unsacculated colon of the dog, which allows for rapid transit and frequent defecation, may also explain the lack of gene expression differences due to dietary treatment. Nonetheless, dietary effects on gene expression were more pronounced in senior dogs (22/32 genes) as compared to young adult dogs (10/32 genes), indicating that an aged colon is more likely to be influenced by dietary characteristics.

**Table 7:** Differentially expressed cell growth and development-, cell signaling and signal transduction-, and immune and stress response-associated genes in colonic mucosa of senior and young adult dogs fed an animal protein-based diet vs. plant protein–based diet.

			Fold change	
Functional classification	Gene name	Gene symbol	Senior	Young
**Cell growth and development**				
Cell cycle	MIND kinetochore complex component	MIS12	1.59	
Cell cycle	Septin 4 isoform 2	SEPT4	1.51	
Proliferation	Bone morphogenetic protein 6	BMP6		2.06
Proliferation	Potassium channel tetramerisation domain containing 10	KCTD10		1.64
Wound healing	Connective tissue growth factor	CTGF	−4.41	
Cytoskeleton	Spectrin repeat containing, nuclear envelope 2	SYNE2	−1.80	
**Cell signaling and signal transduction**				
MAPK pathway	Phosphodiesterase 6H, cGMP-specific, cone, gamma	PDE6H	−2.43	
Neuroendocrine signaling	Secretogranin 1/chromogranin B	CHGB	−2.30	
Signal transduction	SAR1 gene	IQGAP1	−5.68	
Signal transduction	ADP-ribosylation factor-like 6	ARL6		−2.21
**Immune and stress response**				
Immune response	Secretoglobin, family 1A, member 1	SCGB1A1	−9.23	
Immune response	Serum amyloid A1	SAA1	2.08	
Immune response	Heat shock protein 70kDA protein 1A	HSP70-1A	−4.17	

**Table 8:** Differentially expressed cellular metabolism and protein processing- and transcription/translation-associated genes in colonic mucosa of senior and young adult dogs fed an animal protein-based diet vs. plant protein–based diet.

			Fold change	
Functional classification	Gene name	Gene symbol	Senior	Young
**Cellular metabolism and protein processing**				
Glucose metabolism	Glucose phosphate isomerase	GPI	2.20	
Sulfur metabolism	Molybdenum cofactor synthesis 2	MOCS2	1.79	
Hydrolysis	Abhydrolase domain containing 3	ABHD3	1.53	
TCA cycle	Dihydrolipoamide S-succinyltransferase	DLST		1.85
Endosome transport	Early endosome antigen 1	EEA1		−3.34
Protein folding	Serologically defined colon cancer antigen 10	SDCCAG10		−1.78
Protein phosphorylation	PCTAIRE protein kinase 1	PCTK1		1.99
Protein phosphorylation	arginine/serine-rich coiled-coil 1	RSRC1		−1.64
Proteolysis	Dipeptidase 2 (metallopeptidase M20 family)	CNDP2	2.34	
Endocytosis	Formin binding protein 1	FNBP1	1.60	
Membrane traffic	SEC16 homolog A	SEC16A		2.19
**Transcription-Translation**				
Transcription	Mediator complex subunit 13-like	THRAP2	2.59	
Transcription	Nuclear receptor co-repressor 1	NCOR1	2.33	
RNA processing	WW domain binding protein 11	WBP11	−1.51	
DNA repair	BRCA1 associated RING domain 1	BARD1	1.64	
DNA repair	Casein kinase 1, delta	CSNK1D		2.09
**Miscellaneous and unknown**				
Insulin secretion	Family with sequence similarity 3, member B	FAM3B	1.67	
Reproduction	UBX domain containing 6	UBXD6	1.57	
Unknown	Transmembrane protein 205	TMEM205	−1.72	

The SCGB1A1 gene encoding secretoglobin was greatly down-regulated (9.23 fold) in senior dogs consuming APB as compared to senior dogs consuming PPB. Secretoglobin is suggested to play a protective role against inflammation, oxidative damage, and carcinogenesis [75]. Therefore, its down-regulation may implicate a diet containing high fat and low fiber concentrations and low protein digestibility, leading to increased colonic ammonia concentrations (e.g., the APB diet fed in this study) with decreased colonic defensive mechanisms against inflammation and oxidative stress, especially for senior dogs. Likewise, heat shock protein 70 (Hsp70-1A), a molecular chaperone involved in homeostatic regulation of immune and stress responses [76], was also greatly down-regulated (4.17 fold), while SAA1, an acute phase protein, was up-regulated (2.08 fold) in senior dogs consuming APB vs. PPB. It was interesting to note that young adult dogs consuming APB had increased expression of BMP6 gene. This gene is a member of the transforming growth factor β family and is involved with cartilage and skeletal formation [77], but it may modulate immune responses by decreasing proliferation of B-cells and by inducing apoptosis of activated memory B-cells [78]. The implication of increased BMP6 expression in the colon of young adult dogs consuming a high-fat and low-fiber diet is not clear at this time [78].

It was unexpected that expression of CTGF, a gene involved in cellular wound healing [19], was down-regulated (4.41 fold) in senior dogs consuming APB compared with those consuming PPB. The increased colonic concentrations of ammonia, as previously observed in dogs consuming APB [5], has been associated with increased epithelial damage [14], and therefore, CTGF gene expression may be expected to be increased to repair cellular damage. The molecular link between colonic ammonia concentrations and CTGF expression has not been made previously; however, it is possible, but remains to be elucidated, that luminal ammonia decreases cellular repair activity in response to epithelial damage, which may be more prominent in senior dogs. Further research is required to study diet-induced alterations in colonic gene expression by using diets containing greater compositional differences.

In conclusion, the current experiment used microarray technology to identify global changes in colonic gene expression induced by age and diet. The majority of genes were altered by age although a relatively small number of genes were also affected by diet. In particular, aged colonic mucosa had an up-regulation of genes associated with cell proliferation and a down-regulation of genes associated with apoptosis and differentiation, highlighting potential genes and pathways that may be responsible for the predisposition of diseases in the aged colon. Aged colonic mucosa also appeared to have an up-regulation of genes associated with inflammation and stress response and a down-regulation of genes associated with defensive mechanisms. Up-regulation of genes related to cellular metabolism in the aged colon may indicate an elevated metabolic rate in the colonic epithelium. Therefore, our results provide molecular insight pertaining to the aged colon and its predisposition to dysfunction and disease. These data have highlighted metabolic pathways that are altered in the aged colon, many of which may aid in future research pertaining to age-associated changes in colonic physiology and disease risk, and dietary strategies to limit their progression.

## Materials and Methods

### Animals, diets and experimental design

All animal care, handling, and sampling procedures are detailed in Kuzmuk et al. [5] and all experimental procedures were approved by the University of Illinois Institutional Animal Care and Use Committee (IACUC #02056) prior to the initiation of the experiment. Briefly, 12 senior (average age = 11.1 y old at baseline; Kennelwood Inc., Champaign, IL) and 12 young (8 wk old at baseline; Marshall Farms USA, Inc., North Rose, NY) female beagles were randomly allotted to 2 dietary treatments and fed for 12 months. Dietary treatments were reported previously [5], [15]. In short, one diet was an animal protein-based diet (APB) that was formulated to contain 28.0% CP, 22.6% fat, and 4.8% total dietary fiber (TDF) with highly digestible animal-derived ingredients. The other diet was primarily a plant protein-based diet (PPB) that was formulated to contain 25.5% CP, 11.2% fat, and 15.2% TDF with moderately digestible plant-derived ingredients. Both diets were formulated to meet or exceed all nutrient requirements for canine growth according to the Association of American Feed Control Officials [79]. Young dogs were fed *ad libitum* to allow for adequate growth and maintained a healthy body condition score (5/9 to 6/9), while senior dogs were fed to maintain baseline body weight but were likely to have a slightly variable body condition score (3/9 to 7/9) throughout the experiment. All dogs were individually housed in kennels (1.1×0.9 m) in temperature-controlled rooms with a 12-h light∶12-h dark cycle at the Edward R. Madigan Laboratory on the University of Illinois campus.

### Sample collection and RNA extraction

After 12 months of experiment, dogs were fasted for 12 h and euthanized using a lethal dose (130 mg/kg body weight) of sodium pentobarbital (Euthasol®, Virbac Corp., Fort Worth, TX). Colon tissue (midpoint) was immediately collected, flash frozen using liquid nitrogen, and stored at −80°C. A small amount of colon tissue from the 6 females from each age group was then placed in RNAlater ICE (Ambion, Austin, TX), thawed at −20°C, and mucosa was scraped for RNA extraction. Total cellular RNA was isolated from all mucosa samples using Trizol (Invitrogen, Carlsbad, CA). RNA concentration was measured using a ND-1000 spectrophotometer (Nanodrop Technologies, Wilmington, DE). RNA integrity was verified on a 1.2% denaturing agarose gel.

### Microarray procedure and data analyses

The procedures for microarray data analyses were described previously by Swanson et al. [11]. Briefly, the prepared RNA samples were hybridized to Affymetrix GeneChip® Canine Genome Arrays (Affymetrix, Santa Clara, CA). After hybridization, chips were washed and stained with streptavidin-conjugated phycoerythrin dye (Invitrogen) enhanced with biotinylated goat anti-streptavidin antibody (Vector Laboratories, Burlingame, CA) utilizing an Affymetrix GeneChip® Fluidics Station 450 and GeneChip® Operating Software. Images were then scanned using an Affymetrix GeneChip® Scanner 3000. Of the 23,836 probe sets on the array, 14,217 probe sets were expressed in the colonic mucosa and were assessed for gene expression changes due to age and diet. Heat maps were generated and MetaCore (GeneGo, Inc., St. Joseph, MI) was used to build gene networks and interpret microarray data. Functional attribution was made by the database SOURCE (http://source.stanford.edu) [80]. All microarray data have been deposited in the Gene Expression Omnibus (GEO) repository at the National Center for Biotechnology Information (NCBI) archives (http://www.ncbi.nlm.nih.gov/geo) under accession #GSE20557.

### Statistical analysis

To assess inter-animal variation, colonic mucosa samples were not pooled in this experiment. Therefore, each animal was analyzed as an individual experimental unit. Differential expression of the microarray data was evaluated using the limma package [81]. A linear model for the four age x diet groups was fit for each probe set. Differences between groups were then extracted from the model as contrasts. An empirical Bayes “shrinkage” method was employed on the standard errors to improve power for small sample sizes [81]. Lastly, multiple test correction of *p*-values was done using the false discovery rate (FDR) method [82]. Gene transcripts having >1.5-fold change and FDR <0.10 were considered significantly different.

## References

[1] O'Keefe SJ (2008). Nutrition and colonic health: the critical role of the microbiota.. Curr Opin Gastroenterol.

[2] Hoops TC, Traber PG (1997). Molecular pathogenesis of colorectal cancer.. Hematol Oncol Clin North Am.

[3] Commane DM, Arasaradnam RP, Mills S, Mathers JC, Bradburn M (2009). Diet, ageing and genetic factors in the pathogenesis of diverticular disease.. World J Gastroenterol.

[4] Kleinschmidt S, Meneses F, Nolte I, Hewicker-Trautwein M (2008). Distribution of mast cell subtypes and immune cell populations in canine intestines: Evidence for age-related decline in T cells and macrophages and increase of IgA-positive plasma cells.. Res Vet Sci.

[5] Kuzmuk KN, Swanson KS, Tappenden KA, Schook LB, Fahey GC (2005). Diet and age affect intestinal morphology and large bowel fermentative end-product concentrations in senior and young adult dogs.. J Nutr.

[6] Swanson KS, Schook LB, Fahey GC (2003). Nutritional genomics: implications for companion animals.. J Nutr.

[7] Notterman DA, Alon U, Sierk AJ, Levine AJ (2001). Transcriptional gene expression profiles of colorectal adenoma, adenocarcinoma, and normal tissue examined by oligonucleotide arrays.. Cancer Res.

[8] Lefevre M, Wiles JE, Zhang X, Howard LR, Gupta S (2008). Gene expression microarray analysis of the effects of grape anthocyanins in mice: a test of a hypothesis-generating paradigm.. Metabolism.

[9] Lee CK, Klopp RG, Weindruch R, Prolla TA (1999). Gene expression profile of aging and its retardation by caloric restriction.. Science.

[10] Lee D, Lee K, Choi J, Hyun J, Lee E (2009). cDNA microarray analysis of differential gene expression in boar testes during the prepubertal period.. Asian-Aust J Anim Sci.

[11] Swanson KS, Vester BM, Apanavicius CJ, Kirby NA, Schook LB (2009). Implications of age and diet on canine cerebral cortex transcription.. Neurobiol Aging.

[12] Middelbos IS, Vester BM, Karr-Lilienthal LK, Schook LB, Swanson KS (2009). Age and diet affect gene expression profile in canine skeletal muscle.. PLoS One.

[13] Swanson KS, Belsito KR, Vester BM, Schook LB (2009). Adipose tissue gene expression profiles of healthy young adult and geriatric dogs.. Arch Anim Nutr.

[14] Macfarlane GT, Cummings JH, Phillips SF, Pembertin JH, Shorter RG (1991). The colonic flora, fermentation, and large bowel digestive function.. The Large Intestine: Physiology, Pathophysiology, and Disease.

[15] Swanson KS, Kuzmuk KN, Schook LB, Fahey GC (2004). Diet affects nutrient digestibility, hematology, and serum chemistry of senior and weanling dogs.. J Anim Sci.

[16] Lee HM, Greeley GH, Englander EW (2001). Age-associated changes in gene expression patterns in the duodenum and colon of rats.. Mech Ageing Dev.

[17] Vester BM, Liu KJ, Keel TL, Graves TK, Swanson KS (2009). In utero and postnatal exposure to a high-protein or high-carbohydrate diet leads to differences in adipose tissue mRNA expression and blood metabolites in kittens.. Br J Nutr.

[18] Gamlem H, Nordstoga K, Glattre E (2008). Canine neoplasia - Introductory paper.. Apmis Suppl.

[19] Igarashi A, Okochi H, Bradham DM, Grotendorst GR (1993). Regulation of connective tissue growth factor gene expression in human skin fibroblasts and during wound repair.. Mol Biol Cell.

[20] Lin BR, Chang CC, Che TF, Chen ST, Chen RJ (2005). Connective tissue growth factor inhibits metastasis and acts as an independent prognostic marker in colorectal cancer.. Gastroenterology.

[21] Deng YZ, Chen PP, Wang Y, Yin D, Koeffler HP (2007). Connective tissue growth factor is overexpressed in esophageal squamous cell carcinoma and promotes tumorigenicity through beta-catenin-T-cell factor/Lef signaling.. J Biol Chem.

[22] Yang F, Tuxhorn JA, Ressler SJ, McAlhany SJ, Dang TD (2005). Stromal expression of connective tissue growth factor promotes angiogenesis and prostate cancer tumorigenesis.. Cancer Res.

[23] Xie D, Nakachi K, Wang H, Elashoff R, Koeffler HP (2001). Elevated levels of connective tissue growth factor, WISP-1, and CYR61 in primary breast cancers associated with more advanced features.. Cancer Res.

[24] Wu CC, Shyu RY, Chou JM, Jao SW, Chao PC (2006). RARRES1 expression is significantly related to tumour differentiation and staging in colorectal adenocarcinoma.. Eur J Cancer.

[25] Stossel TP, Condeelis J, Cooley L, Hartwig JH, Noegel A (2001). Filamins as integrators of cell mechanics and signalling.. Nat Rev Mol Cell Biol.

[26] Zhou X, Tian F, Sandzen J, Cao R, Flaberg E (2007). Filamin B deficiency in mice results in skeletal malformations and impaired microvascular development.. Proc Natl Acad Sci U S A.

[27] Kato T, Satoh S, Okabe H, Kitahara O, Ono K (2001). Isolation of a novel human gene, MARKL1, homologous to MARK3 and its involvement in hepatocellular carcinogenesis.. Neoplasia.

[28] Pederson L, Ruan M, Westendorf JJ, Khosla S, Oursler MJ (2008). Regulation of bone formation by osteoclasts involves Wnt/BMP signaling and the chemokine sphingosine-1-phosphate.. Proc Natl Acad Sci U S A.

[29] Pal R, Khanna A (2006). Role of smad- and wnt-dependent pathways in embryonic cardiac development.. Stem Cells Dev.

[30] Lee RH, Iioka H, Ohashi M, Iemura S, Natsume T (2007). XRab40 and XCullin5 form a ubiquitin ligase complex essential for the noncanonical Wnt pathway.. EMBO J.

[31] Voutsadakis IA (2008). The ubiquitin-proteasome system in colorectal cancer.. Biochim Biophys Acta.

[32] Xu Y, Pasche B (2007). TGF-beta signaling alterations and susceptibility to colorectal cancer.. Hum Mol Genet.

[33] Zhang Y, Feng XH, Derynck R (1998). Smad3 and Smad4 cooperate with c-Jun/c-Fos to mediate TGF-beta-induced transcription.. Nature.

[34] Pearson G, Robinson F, Beers Gibson T, Xu BE, Karandikar M (2001). Mitogen-activated protein (MAP) kinase pathways: regulation and physiological functions.. Endocr Rev.

[35] Kiss-Toth E, Bagstaff SM, Sung HY, Jozsa V, Dempsey C (2004). Human tribbles, a protein family controlling mitogen-activated protein kinase cascades.. J Biol Chem.

[36] Mori M, Shiraishi T, Tanaka S, Yamagata M, Mafune K (1999). Lack of DMBT1 expression in oesophageal, gastric and colon cancers.. Br J Cancer.

[37] Mollenhauer J, Helmke B, Muller H, Kollender G, Krebs I (2002). An integrative model on the role of DMBT1 in epithelial cancer.. Cancer Detect Prev.

[38] Pyronnet S, Bousquet C, Najib S, Azar R, Laklai H (2008). Antitumor effects of somatostatin.. Mol Cell Endocrinol.

[39] Tulassay Z (1998). Somatostatin and the gastrointestinal tract.. Scand J Gastroenterol Suppl.

[40] Harding MA, Theodorescu D (2007). RhoGDI2: a new metastasis suppressor gene: discovery and clinical translation.. Urol Oncol.

[41] Shouse GP, Cai X, Liu X (2008). Serine 15 phosphorylation of p53 directs its interaction with B56gamma and the tumor suppressor activity of B56gamma-specific protein phosphatase 2A.. Mol Cell Biol.

[42] Wang Y, Devereux W, Stewart TM, Casero RA (1999). Cloning and characterization of human polyamine-modulated factor-1, a transcriptional cofactor that regulates the transcription of the spermidine/spermine N(1)-acetyltransferase gene.. J Biol Chem.

[43] Luk GD (1990). Polyamines in intestinal growth.. Biochem Soc Trans.

[44] Hameed S, Dhillo WS, Bloom SR (2009). Gut hormones and appetite control.. Oral Dis.

[45] Schjoldager BT, Mortensen PE, Christiansen J, Orskov C, Holst JJ (1989). GLP-1 (glucagon-like peptide 1) and truncated GLP-1, fragments of human proglucagon, inhibit gastric acid secretion in humans.. Dig Dis Sci.

[46] Willms B, Werner J, Holst JJ, Orskov C, Creutzfeldt W (1996). Gastric emptying, glucose responses, and insulin secretion after a liquid test meal: effects of exogenous glucagon-like peptide-1 (GLP-1)-(7-36) amide in type 2 (noninsulin-dependent) diabetic patients.. J Clin Endocrinol Metab.

[47] Drucker DJ (2003). Glucagon-like peptides: regulators of cell proliferation, differentiation, and apoptosis.. Mol Endocrinol.

[48] Ghatei MA, Goodlad RA, Taheri S, Mandir N, Brynes AE (2001). Proglucagon-derived peptides in intestinal epithelial proliferation: glucagon-like peptide-2 is a major mediator of intestinal epithelial proliferation in rats.. Dig Dis Sci.

[49] James SP (1993). The gastrointestinal mucosal immune system.. Dig Dis.

[50] Watson AJ (2006). An overview of apoptosis and the prevention of colorectal cancer.. Crit Rev Oncol Hematol.

[51] Kone BC, Kuncewicz T, Zhang W, Yu ZY (2003). Protein interactions with nitric oxide synthases: controlling the right time, the right place, and the right amount of nitric oxide.. Am J Physiol Renal Physiol.

[52] Yang GY, Taboada S, Liao J (2009). Induced nitric oxide synthase as a major player in the oncogenic transformation of inflamed tissue.. Methods Mol Biol.

[53] Keklikoglu N, Koray M, Kocaelli H, Akinci S (2008). iNOS expression in oral and gastrointestinal tract mucosa.. Dig Dis Sci.

[54] Rodriguez-Cabezas ME, Galvez J, Lorente MD, Concha A, Camuesco D (2002). Dietary fiber down-regulates colonic tumor necrosis factor alpha and nitric oxide production in trinitrobenzenesulfonic acid-induced colitic rats.. J Nutr.

[55] Chang W, Ma L, Lin L, Gu L, Liu X (2009). Identification of novel hub genes associated with liver metastasis of gastric cancer.. Int J Cancer.

[56] Kiyokawa N, Sekino T, Matsui T, Takenouchi H, Mimori K (2004). Diagnostic importance of CD179a/b as markers of precursor B-cell lymphoblastic lymphoma.. Mod Pathol.

[57] Henderson NC, Sethi T (2009). The regulation of inflammation by galectin-3.. Immunol Rev.

[58] Xu L, Badolato R, Murphy WJ, Longo DL, Anver M (1995). A novel biologic function of serum amyloid A. Induction of T lymphocyte migration and adhesion.. J Immunol.

[59] Urieli-Shoval S, Cohen P, Eisenberg S, Matzner Y (1998). Widespread expression of serum amyloid A in histologically normal human tissues. Predominant localization to the epithelium.. J Histochem Cytochem.

[60] DeLeve LD, Kaplowitz N (1991). Glutathione metabolism and its role in hepatotoxicity.. Pharmacol Ther.

[61] Pool-Zobel B, Veeriah S, Bohmer FD (2005). Modulation of xenobiotic metabolising enzymes by anticarcinogens – focus on glutathione S-transferases and their role as targets of dietary chemoprevention in colorectal carcinogenesis.. Mutat Res.

[62] Velazquez OC, Lederer HM, Rombeau JL (1997). Butyrate and the colonocyte. Production, absorption, metabolism, and therapeutic implications.. Adv Exp Med Biol.

[63] Fitch MD, Fleming SE (1999). Metabolism of short-chain fatty acids by rat colonic mucosa in vivo.. Am J Physiol.

[64] Saksena S, Theegala S, Bansal N, Gill RK, Tyagi S (2009). Mechanisms Underlying Modulation of Monocarboxylate Transporter 1 (MCT1) by Somatostatin in Human Intestinal Epithelial Cells.. Am J Physiol Gastrointest Liver Physiol.

[65] Williams EA, Coxhead JM, Mathers JC (2003). Anti-cancer effects of butyrate: use of micro-array technology to investigate mechanisms.. Proc Nutr Soc.

[66] Scharlau D, Borowicki A, Habermann N, Hofmann T, Klenow S (2009). Mechanisms of primary cancer prevention by butyrate and other products formed during gut flora-mediated fermentation of dietary fibre.. Mutat Res.

[67] Hunt ND, Hyun DH, Allard JS, Minor RK, Mattson MP (2006). Bioenergetics of aging and calorie restriction.. Ageing Res Rev.

[68] Van Schaftingen E, Detheux M, Veiga da Cunha M (1994). Short-term control of glucokinase activity: role of a regulatory protein.. FASEB J.

[69] Eklof V, Van Guelpen B, Hultdin J, Johansson I, Hallmans G (2008). The reduced folate carrier (RFC1) 80G>A and folate hydrolase 1 (FOLH1) 1561C>T polymorphisms and the risk of colorectal cancer: a nested case-referent study.. Scand J Clin Lab Invest.

[70] Choi SW, Friso S, Dolnikowski GG, Bagley PJ, Edmondson AN (2003). Biochemical and molecular aberrations in the rat colon due to folate depletion are age-specific.. J Nutr.

[71] Velayos-Baeza A, Vettori A, Copley RR, Dobson-Stone C, Monaco AP (2004). Analysis of the human VPS13 gene family.. Genomics.

[72] Tardy C, Sabourdy F, Garcia V, Jalanko A, Therville N (2009). Palmitoyl protein thioesterase 1 modulates tumor necrosis factor alpha-induced apoptosis.. Biochim Biophys Acta.

[73] Ozawa H, Takata K (1995). The granin family–its role in sorting and secretory granule formation.. Cell Struct Funct.

[74] Vogel VG, McPherson RS (1989). Dietary epidemiology of colon cancer.. Hematol Oncol Clin North Am.

[75] Mukherjee AB, Zhang Z, Chilton BS (2007). Uteroglobin: a steroid-inducible immunomodulatory protein that founded the Secretoglobin superfamily.. Endocr Rev.

[76] Henderson B (2009). Integrating the cell stress response: a new view of molecular chaperones as immunological and physiological homeostatic regulators.. Cell Biochem Funct.

[77] Chen D, Zhao M, Harris SE, Mi Z (2004). Signal transduction and biological functions of bone morphogenetic proteins.. Front Biosci.

[78] Kersten C, Sivertsen EA, Hystad ME, Forfang L, Smeland EB (2005). BMP-6 inhibits growth of mature human B cells; induction of Smad phosphorylation and upregulation of Id1.. BMC Immunol.

[79] AAFCO (2004). Official publication.

[80] Diehn M, Sherlock G, Binkley G, Jin H, Matese JC (2003). SOURCE: a unified genomic resource of functional annotations, ontologies, and gene expression data.. Nucleic Acids Res.

[81] Smyth GK (2004). Linear Models and Empirical Bayes Methods for Assessing Differential Expression in Microarray Experiments. Statistical Applications in Genetics and Molecular Biology.

[82] Benjamini Y, Hochberg Y (1995). Controlling the False Discovery Rate - a Practical and Powerful Approach to Multiple Testing.. J Roy Stat Soc Ser.

